# Lensless light-field imaging through diffuser encoding

**DOI:** 10.1038/s41377-020-00380-x

**Published:** 2020-08-19

**Authors:** Zewei Cai, Jiawei Chen, Giancarlo Pedrini, Wolfgang Osten, Xiaoli Liu, Xiang Peng

**Affiliations:** 1 Institut für Technische Optik, Universität Stuttgart, Pfaffenwaldring 9, 70569 Stuttgart, Germany; 2 College of Physics and Optoelectronic Engineering, Shenzhen University, 518060 Guangdong, Shenzhen China

**Keywords:** Optical sensors, Imaging and sensing

## Abstract

Microlens array-based light-field imaging has been one of the most commonly used and effective technologies to record high-dimensional optical signals for developing various potential high-performance applications in many fields. However, the use of a microlens array generally suffers from an intrinsic trade-off between the spatial and angular resolutions. In this paper, we concentrate on exploiting a diffuser to explore a novel modality for light-field imaging. We demonstrate that the diffuser can efficiently angularly couple incident light rays into a detected image without needing any lens. To characterize and analyse this phenomenon, we establish a diffuser-encoding light-field transmission model, in which four-dimensional light fields are mapped into two-dimensional images via a transmission matrix describing the light propagation through the diffuser. Correspondingly, a calibration strategy is designed to flexibly determine the transmission matrix, so that light rays can be computationally decoupled from a detected image with adjustable spatio-angular resolutions, which are unshackled from the resolution limitation of the sensor. The proof-of-concept approach indicates the possibility of using scattering media for lensless four-dimensional light-field recording and processing, not just for two- or three-dimensional imaging.

## Imaging: Lensless light-field imaging lays foundations for new applications

By replacing lenses with a diffuser in light-field imaging, scientists could expand its use in applications ranging from light-field microscopy and synthetic aperture imaging to visual odometry. Microlens array-based light-field imaging is one of the most commonly used technologies to record high-dimensional optical images. However, they suffer from a trade-off between the spatial and angular resolutions. Xiaoli Liu and colleagues from Shenzhen University in China, in collaboration with researchers from the University of Stuttgart in Germany, have now developed a novel technique that uses a diffuser to angularly couple incident light rays into a detected image without the need for a lens. The diffuser allows each sub-beam emitted by a point source to form a distinguishable sub-image covering a region on the sensor and could lead to lensless four-dimensional light-field imaging.

## Introduction

Conventional photography forms a two-dimensional image on a sensor, leading to loss of angular information. In contrast, light-field imaging can detect both spatial and angular information^1,2^. The angular information offers peculiar capabilities over conventional imaging, such as viewpoint shifting, post-capture refocusing, depth sensing, and depth-of-field extension. Consequently, light-field imaging has a growing range of applications involving light-field microscopy^3–7^, synthetic aperture imaging^8,9^, visual odometry and localization^10,11^, and many others.

For light-field imaging, a primary and critical step is how to effectively record angular-resolved light fields. According to the number of image sensors and exposures used, light-field imaging can be mainly classified into three categories. One is using multiple image sensors with a single exposure to simultaneously capture light-field samples from different viewpoints^5,8,12^. Such a system usually consists of a large number of cameras, which are bulky and expensive and thus not suitable for practical applications. Alternatively, light-field samples can be obtained using a single sensor and multiple exposures^1,2,13^. This approach can record light fields with high spatial and angular resolutions, but it is time-consuming and thus unsuitable for dynamic scenes.

The third approach uses a single image sensor with a single exposure to encode four-dimensional spatio-angular information into a two-dimensional detected image. More than a century ago, the concept of plenoptic cameras by adding a pinhole array or microlens array was proposed^14,15^. Currently, microlens array-based plenoptic cameras are commonly used for light-field imaging^16–18^, such as the commercially available products, Lytro and Raytrix. These devices can record unambiguous light fields in which a pixel corresponds to a single light ray and can conceptually be thought of as classical light-field imaging. However, classical light-field imaging involves a trade-off between the spatial and angular resolutions; the spatial resolution is in general tens to hundreds times smaller than the number of pixels used. In recent years, alternative techniques have been developed to encode a light ray on multiple pixels through modulation masks (e.g., attenuation masks^19–21^ and diffuser plates^22^), avoiding the resolution trade-off. A light field is no longer recorded directly but computationally retrieved from a detected image, which can be referred to as computational light-field imaging.

In this paper, we propose a novel modality for computational light-field imaging by using a diffuser as an encoder, without needing any lens. Through the diffuser, each sub-beam directionally emitted by a point source in the detectable field-of-view forms a distinguishable sub-image that covers a specific region on the sensor. These sub-images are combined into a unique pseudorandom pattern corresponding to the response of the system to the point source. Consequently, the system has the capability of encoding a light-field incident onto the diffuser. We establish a diffuser-encoding light-field transmission model to characterize the mapping of four-dimensional light fields to two-dimensional images, where a pixel collects and integrates contributions from different sub-beams. With the aid of the optical properties of the diffuser encoding, the light-field transmission matrix can be flexibly calibrated through a point source generated pattern. As a result, light fields are computationally reconstructed with adjustable spatio-angular resolutions, avoiding the resolution limitation of the sensor. Being significantly different from the existing approaches using diffusers for lensless two- or three-dimensional imaging^23–26^, our imaging modality provides a proof-of-concept approach in which scattering media can be exploited for recording high-dimensional optical signals, modewith which the intrinsic mechanism of light propagation can be further explored.

## Results

### Diffuser encoding

**Fig. 1:**
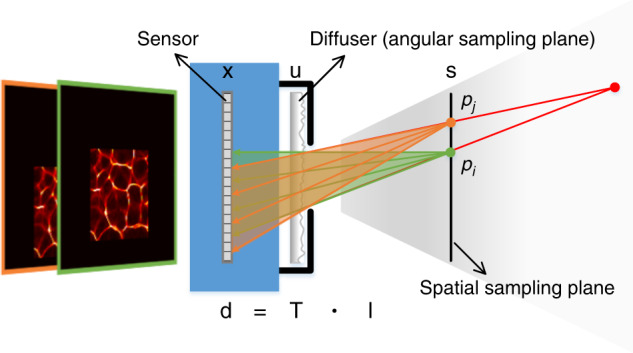
Schematic diagram of lensless light-field imaging through diffuser encoding. Here, **s**, **u**, and **x** are coordinates on the spatial sampling plane, angular sampling plane, and sensor plane, respectively; **d** is the vector describing the sensor detection; **l** is the vector describing the objective light field; and **T** is the light-field transmission matrix

The diffuser used in the system (see Fig. 1) is a thin transparent phase plate with a statistically varying surface distribution. Through the diffuser, a temporally incoherent point source in the detectable field-of-view generates a high-contrast pseudorandom pattern on the sensor, as shown by the green and orange wireframes in Fig. 1. Consistent with imaging through scattering media, the objective information of the point source is encoded, rather than lost, in the pattern. We assume that the light-beam emitted by the point source can be manipulated and angularly divided into a number of thin sub-beams. Each sub-beam illuminates a small region of the diffuser surface and forms a sub-image that corresponds to a segment of the pattern. These sub-images are different from each other because of the random roughness distribution of the diffuser surface. With appropriate manipulation, these sub-images do not overlap each other and exactly constitute the pattern. The validity of the assumption can be verified through experiments. In this situation, the elementary sub-beams, represented by their centre light rays, are angularly encoded in the pattern, as illustrated by the green and orange lines with arrows in Fig. 1. Thus, the light field of the point source is encoded by the diffuser.

Similar to imaging through scattering media^27,28^, the response of the diffuser to a point source is lateral shift invariant. A lateral shift of the point source results in a translation of the corresponding pattern. This means that each point source in a plane parallel to the diffuser plane generates a unique pseudorandom pattern. Therefore, a light ray emitted by any in-plane point source and incident onto the diffuser along a specific direction corresponds to a unique sub-image. This phenomenon brings two benefits. One is that encoding a light ray depends on the position and angle at which the light ray is incident onto the diffuser regardless of whether the point source is in the parallel plane. As illustrated in Fig. 1, the diffuser plays an equivalent role with light rays emitted by an out-of-plane point source (red point) and these by in-plane point sources (green and orange points). In other words, all light rays in the detectable field-of-view can be uniquely encoded, enabling lensless light-field imaging. The other benefit is that the light field can be represented via a two-plane parameterization. The diffuser plane associated with the incident directions of light rays is defined as the angular sampling plane. Another plane parallel to the diffuser plane is associated with the positions of point sources and defined as the spatial sampling plane. Consequently, the light field can be represented as *l*(**s**, **u**), where *l* is the radiance and **s** = (*s*, *t*)^T^ and **u** = (*u*, *v*)^T^ are spatial and angular coordinates, respectively, as labelled in Fig. 1.

First, we consider only one point source in the spatial sampling plane. Light rays emitted by a temporally incoherent point source, *p*_*i*_, are incoherent with each other. Thus, the pseudorandom pattern generated by this point source is a linear combination of the non-overlapped sub-images corresponding to these light rays. This process can be modelled as a matrix-vector multiplication:

1 $${\mathbf{f}}_i = {\mathbf{H}}_i{\mathbf{g}}_i$$

where **f**_*i*_ is a vector describing the pattern, **g**_*i*_ is a vector describing the single-point light field, ${\mathbf{H}}_i = \left( {{\mathbf{t}}_{i,1},{\mathbf{t}}_{i,2}, \cdots ,{\mathbf{t}}_{i,n}} \right)$ is a matrix representing the light-field transmission of the point source through the diffuser, and **t**_*i*,*j*_ denotes a vector associated with the sub-image corresponding to the *j*-th light ray of the *i*-th point source.

The detected image of a scene containing multiple point sources on the spatial sampling plane is a linear combination of all pseudorandom patterns, namely the contributions from all light rays in the detectable field-of-view. As a result, lensless light-field imaging through diffuser encoding can be modelled as

2 $${\mathbf{d}} = {\mathbf{Tl}}$$

where ${\mathbf{d}}={\sum\nolimits}_{i}{\mathbf{f}}_{i}$ is a vector describing the sensor detection, ${\mathbf{l}} = \left( {{\mathbf{g}}_1,{\mathbf{g}}_2, \cdots ,{\mathbf{g}}_k} \right)^{\mathrm{T}}$ is a vector describing the multi-point light field to be reconstructed, and ${\mathbf{T}} = \left( {{\mathbf{H}}_1,{\mathbf{H}}_2, \cdots ,{\mathbf{H}}_k} \right)$ is a matrix representing the imaging system via which the multi-point light field is encoded to form the image. The structures of Eqs. (1) and (2) are schematically shown in Fig. S1 for the case *n* = 16.

Here, we regard **T** as a light-field transmission matrix based on diffuser encoding. The number of non-zero values in a row vector of **T** might be more than 1. This means that a pixel can collect and integrate various contribution components from different light rays incident on the diffuser. This makes the number of light rays larger than the number of pixels used. The modulation of the light field by the diffuser couples more light-field spectrum information to be recorded. Therefore, the lensless imaging system through diffuser encoding can break the resolution limitation of the sensor, allowing light-efficient high-resolution light-field imaging.

We constructed the experimental system and 12 patterns were captured by axially moving the point source. As mentioned in the following section, a point source generated pattern is sufficient to completely determine the light-field transmission matrix. One of the captured patterns can be selected as a base for calibration and reconstruction. The lensless imaging system was demonstrated and analysed for distributed object points and area objects.

### Light-field imaging for distributed object points

**Fig. 2:**
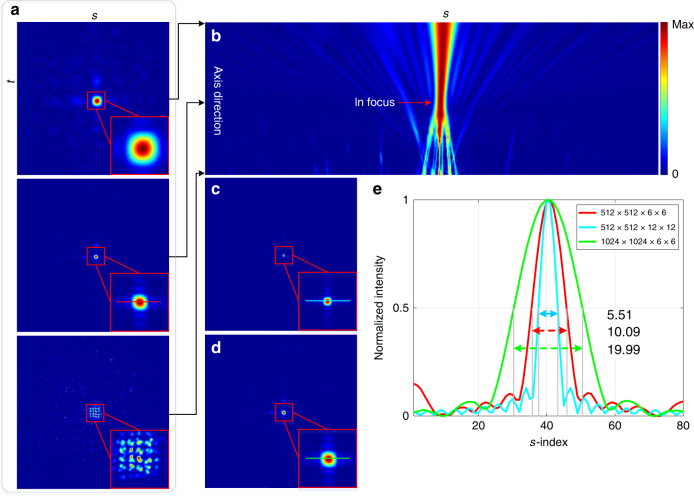
Light-field imaging for a single object point. **a** Three slices (the second one is in-focus) and **b** cross-sectional profile of the focal stack in Resolution 1; **c**, **d** In-focus slices in Resolution 2 and Resolution 3, respectively; **e** Distribution curves of the cross-sections of the in-focus slices with normalized intensity, corresponding to the marked lines in **a**, **c**, and **d**

We used Pattern 6 (see Fig. S4) to calibrate the light-field transmission matrix. The point source corresponding to Pattern 1 was regarded as an object point to be measured. The spatial and angular samplings were selected to be 512 × 512 and 6 × 6, respectively, denoted as Resolution 1 (see Fig. S5). This is equivalent to 9.4 million light rays computationally decoupled from a 0.26-megapixel image. The run time for the reconstruction is nearly 8 min (200 iterations, 2.4 s per iteration). To analyse the reconstruction result, we performed digital refocusing^16,29^ with the reconstructed light-field data to obtain a focal stack. Figure 2a shows three slices of the focal stack, in which the second view is in-focus. The cross-sectional profile of the focal stack is shown in Fig. 2b. It has an hourglass shape, where the position of the waist corresponds to the in-focus depth. Additionally, the profile spreads from the waist to two sides along the axial direction, which is exactly consistent with the light-beam propagation.

The decoupling reconstruction was also performed with light-field resolutions of 512 × 512 × 12 × 12 and 1024 × 1024 × 6 × 6, denoted as Resolution 2 and Resolution 3, respectively. The in-focus slices in Resolution 2 and Resolution 3 are shown in Fig. 2c, d, respectively. The distribution curves of the cross-sections of the three in-focus slices with normalized intensity are shown in Fig. 2e. We used the full width at half maximum of the distribution curves to represent the computationally resolved size of the object point so that the reconstruction performances could be compared quantitatively. Table 1 lists the relevant data of the run times and resolved sizes.

**Table 1:** Relevant data of run times (min) and resolved sizes in different resolutions

	Resolution 1	Resolution 2	Resolution 3
Run time	8.00	32.56	32.85
Resolved size	10.09	5.51	19.99

The run times in Resolution 2 and Resolution 3 are approximately equal and four times that in Resolution 1 and thus are approximately proportional to the total number of decoupled light rays. Although the resolved size in Resolution 3 is twice that in Resolution 1, their real resolved sizes are almost the same since the spatial sampling in the *s*- and *t*-dimensions in Resolution 1 is half of that in Resolution 3. As the physical size of the object point (15 µm pinhole) used is quite small to be solved by the system, increasing the spatial sampling does not have a positive impact on reducing the resolved size, but instead leads to a quadratic increase of the run time. In contrast, increasing the angular sampling can effectively reduce the resolved size, as illustrated by the cases in Resolution 1 and Resolution 2.

**Fig. 3:**
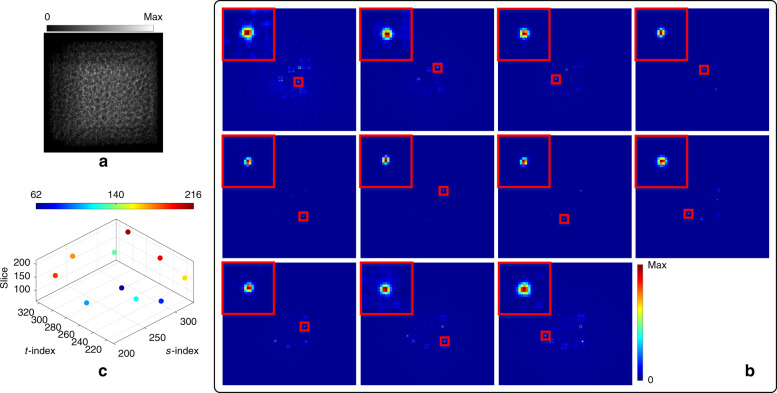
Light-field imaging for multiple sparse object points. **a** Simulated captured image; **b** In-focus slices of the focal stack at different depths, in which the corresponding in-focus object points are marked by red boxes and enlarged on the top left; **c** Spatial distribution of the object points

In the next experiment, Pattern 6 was still regarded as a base, and the other 11 patterns were shifted with random integer pixels (see Fig. S6) and then combined to simulate an experimental scene consisting of multiple object points randomly distributed in the detectable field of view. The simulated captured image is shown in Fig. 3a. According to the above comparison results, the multi-point light field was computationally reconstructed in Resolution 2. By digital refocusing with the reconstructed light field, in-focus slices at different depths, where each object point is located, were obtained (see Fig. 3b). The object points are marked by red boxes; at each in-focus depth, the view simultaneously shows the sharp in-focus point and other blurred defocused points. The reconstructed light field can clearly distinguish different object points at different depths, as illustrated by the spatial distribution in Fig. 3c. Each in-focus object point is enlarged on the top left of each view in Fig. 3b, and the numbers correspond to each captured pattern. The resolved size changes with the axial distance between the measured object point and the point source generating the calibration pattern: the farther the axial distance is, the larger the resolved size.

### Light-field imaging for area objects

**Fig. 4:**
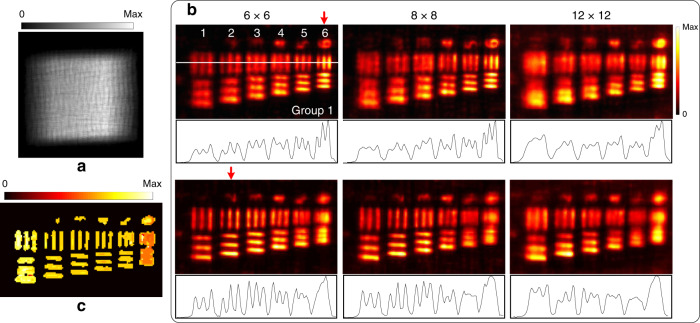
Light-field imaging with different angular samplings. **a** Captured raw image; **b** In-focus slices of the focal stack at different depths indicated by red arrows, and cross-section (labelled by a white line) of each in-focus slice is drawn under each view; **c** Depth map

To compare with the results of distributed object points, a USAF-1951 transmission resolution target was used. The target was tilted with respect to the system, and the investigated area was back-illuminated. A raw image was captured, as shown in Fig. 4a. In this experiment, the spatial sampling (512 × 512) remained unchanged, and different angular samplings (6 × 6, 8 × 8, and 12 × 12) were used for the light-field reconstruction with Pattern 6 as a base. Figure 4b shows every three in-focus slices of the focal stacks corresponding to respective reconstructed light fields at different depths. Cross-sections are also shown under each view. Increasing the angular sampling does not improve the reconstruction results for area objects, which is different compared with the case of distributed object points. By using the focal stack, the depth of the measured object was estimated in the spatial-angular sampling of 512 × 512 × 6 × 6, as shown in Fig. 4c.

**Fig. 5:**
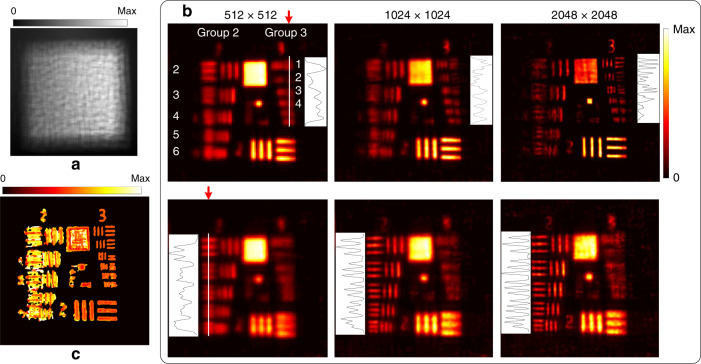
Light-field imaging with different spatial samplings. **a** Captured raw image; **b** In-focus slices of the focal stack at different depths indicated by red arrows, and cross-sections (labeled by the white lines) of each in-focus slice is drawn beside each view; **c** Depth map

To investigate the impact of the spatial sampling on the light-field reconstruction for area objects, we moved the target closer to the system at a distance of 25 mm and illuminated another region of the target. Figure 5a shows the captured raw image. The angular sampling (6 × 6) remained unchanged, and different spatial samplings (512 × 512, 1024 × 1024, and 2048 × 2048) were used to reconstruct light fields. However, using Pattern 6 as a base could not obtain good results. According to the scaling property of imaging through scattering media, we rescaled Pattern 6 to simulate another pattern as generated by a point source located at a different depth (see Fig. S7). The scaled pattern was used as a base to obtain acceptable reconstruction results. The in-focus slices of the focal stacks are shown in Fig. 5b. By near refocusing, Element 3/Group 3 of the resolution target, equivalent to a resolution of 49.6 µm, could be resolved in the highest spatial sampling, while in the lowest spatial sampling the pattern was unrecognizable. By far refocusing, Element 6/Group 2, equivalent to a resolution of 70.1 µm, and Element 4/Group 2, equivalent to a resolution of 88.3 µm, were resolved in the highest and lowest spatial sampling, respectively. Increasing the spatial sampling can improve the resolution for area objects, which is different than the case for distributed object points. The depth of the measured object for the spatial-angular sampling of 2048 × 2048 × 6 × 6 is shown in Fig. 5c.

**Fig. 6:**
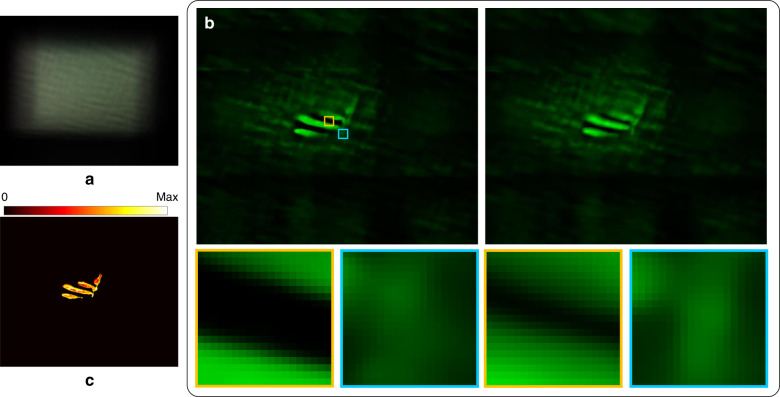
Light-field imaging for a small plant. **a** Captured raw image; **b** In-focus slices of the focal stack at different depths and enlarged segments related to regions marked by the yellow and cyan boxes; **c** Depth map

Finally, we used the experimental data of a small plant^24^ to test our approach. The corresponding raw image is shown in Fig. 6a. A pattern was selected as a base for reconstructing the objective light-field in the spatial-angular sampling of 540 × 640 × 8 × 10. Figure 6b shows two slices of the focal stack and the enlarged views of the regions marked by the orange and cyan boxes. It can be seen that the two slices are in-focus at different depths. The depth map was estimated from the focal stack, as shown in Fig. 6c.

## Discussion

The experimental results show that the proposed approach has different performances regarding spatio-angular sampling and measured objects. We performed singular value decomposition of the light-field transmission matrix **T** to implement an analysis. For convenience, we considered an object composed of a few points in the spatial sampling plane. Each point *p*_*i*_ corresponds to a matrix **H**_*i*_, which can be constructed by shifting the encoding kernel in **H**_0_. These matrices can be combined into a new matrix: ${\mathbf{T}}_{{\mathrm{sub}}} = \left( {{\mathbf{H}}_i,{\mathbf{H}}_j, \cdots } \right)$, which is a submatrix of **T**. We used 5 points spaced by 100 intervals to simulate distributed object points and 20 points with zero intervals to simulate an area object.

**Fig. 7:**
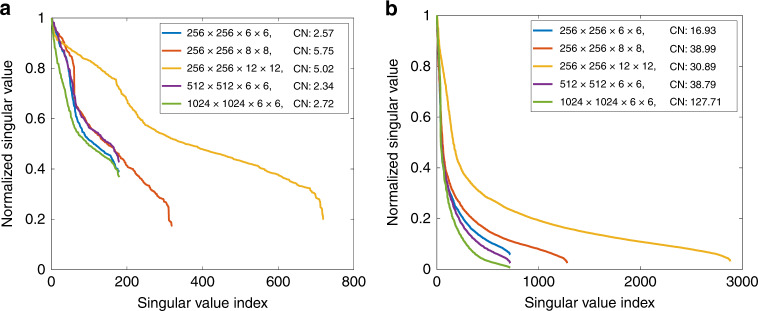
Distributions of singular values of light-field transmission matrices for the simulated objects. **a** Distributed object points and **b** area object in different spatio-angular samplings. CN is the abbreviation for condition number

Figure 7 shows the distributions of normalized singular values. We used the ratio of the maximum singular value to the minimum value to express the condition number. For distributed object points, the approach maintains a lower noise sensitivity than the area object regardless of the spatio-angular sampling. In contrast, for the area object, the singular values decrease more quickly, which means that the hypothesis of the linear independence concerning the transmission matrix becomes weaker and thus that the inverse problem is more ill-posed. In particular, the condition number becomes larger as the spatial sampling increases and more iterations are generally needed in order to obtain convergence (see Fig. S8). Even so, increasing the spatial sampling may make the area object highly resolved. In addition, the analysis indicates that increasing the angular sampling has little effect on the transmission matrix for both distributed object points and the area object. However, in practice, suitably increasing the angular sampling enables more light-field spectrum information to be recorded, resulting in a higher-resolved result for distributed object points. For area objects, however, cross-talk between these spectrum components may not be easily separated, leading to the reduction of the resolution.

A sub-beam emitted by the point source spreads when it propagates to a far axial distance, weakening the hypothesis of the sub-beam behaving as an elementary light ray and thereby leading to a reduction of the lateral resolution at the corresponding depth. The object points far away from the point source could still be recognized with a large resolved size since they were sparsely distributed in the measured volume. When the size of the measured object becomes large, an arbitrary pattern does not guarantee the success of the decoupling reconstruction. This problem can be solved by appropriately scaling the pattern to be applied for the corresponding depth range, although the depth range that can be distinguished for area objects is limited by the specific pattern used.

The improvement of the proposed methodology over the previous work on diffuser-encoding light-field imaging^22^ mainly lies in two aspects. One is that our imaging modality is lensless and thus compact and free of aberration; the other is that the system calibration and decoupling reconstruction become simple and flexible since only one pattern generated by a point source is required. Based on this single-shot lensless light-field imaging modality, light rays, viewpoints, and focal depths can be manipulated and the occlusion problem can be addressed to some extent. This allows us to further investigate the intrinsic mechanism of light-field propagation through the diffuser. It is also possible to transform the diffuser-encoding light-field representation into the Wigner phase space so that the diffraction effect introduced by the internal tiny structure of the diffuser can be taken into account and so that lensless light-field microscopy through diffuser encoding may be developed. Consequently, the proposed approach can be generalized to be used in microscopic imaging, especially fluorescence microscopic imaging, since fluorescent particles are generally sparse in a volume.

## Materials and methods

### Decoupling reconstruction

Equation (2) models a forward imaging process in which the four-dimensional spatio-angular information of a light field, **l**, is coupled into a two-dimensional captured image, **d**, by using the light-field transmission matrix, **T**. To decouple the spatio-angular information from **d** and thereby reconstruct **l**, Eq. (2) must be solved. This is a typical inverse problem; however, as stated above, **T** has more columns than rows, making Eq. (2) an underdetermined problem. Thus, **l** cannot be reconstructed uniquely by directly inverting Eq. (2). If an object is sparse (or sparse in some domains), the inverse problem can be solved via a non-negativity constraint optimization procedure:

3 $${\mathop{\rm{arg}}\limits_{{\mathbf{l}} \ge 0}}\,\min \frac{1}{2}\left\| {{\mathbf{d}} - {\mathbf{Tl}}} \right\|_{2}^{2} \,+\, \tau \left\| {{\Psi}{\mathbf{l}}} \right\|_1$$

The second term in Eq. (3) is a $\ell _1$ regularization, where *τ* is a tuning parameter to adjust the degree of sparsity, and **Ψ** can be selected to be a finite-difference operator or an identity matrix, mapping **l** into a sparse domain.

The light field can be reconstructed by performing the optimization procedure described by Eq. (3) via compressive sensing, provided that the column vectors of **T** are linearly independent. This condition is plausible because each light ray generates a unique pseudorandom sub-image covering a specific region on the sensor. However, as analysed above, linear independence is not necessarily satisfied with various spatio-angular samplings and measured objects. Another necessary condition is to accurately determine the whole elements of **T**, namely to calibrate the light-field transmission matrix. Some techniques, such as interferometry^30,31^ and digital reconstruction^32^, can be used to measure the transmission matrix in imaging through scattering media, but they are suitable only for the cases of coherent illumination. Antipa et al. proposed an approach based on ray tracing to computationally simulate the light-field transmission property of diffuser encoding in incoherent illumination^22^. However, some system parameters, such as the height distribution of the diffuser surface and the distance between the diffuser and image sensor, are mandatory. Moreover, the calibration of the light-field transmission matrix involves the response of the system to each of the millions of light rays in the detectable field-of-view. Such a calibration procedure and extra large-scale matrix operation are impractical for implementing the optimization procedure of Eq. (3).

Fortunately, the lateral shift invariance of the diffuser encoding can be used to reduce the complexity of both the calibration and reconstruction. By placing an aperture close to the diffuser, the support of a pattern generated by a point source, *p*_*i*_, is limited to a specific region on the sensor (see Fig. S3). The detected intensity outside the support is approximately negligible. The support corresponds to a submatrix of **H**_*i*_ with a row range of a non-zero region [*r*_up_, *r*_down_] (see Fig. S1). We regard the submatrix as an encoding kernel of the diffuser acting on the radiant light field of the point source. Similarly, the encoding kernel satisfies the shift invariance. Taking the on-axis point source, *p*_0_, in the spatial sampling plane as a base, the shift invariance of the encoding kernel can be represented as ${\mathbf{H}}_0^{\left[ {r_{{\mathrm{up}}},r_{{\mathrm{down}}}} \right] \times n} = {\mathbf{H}}_i^{\left[ {r_{{\mathrm{up}}} \pm r_{{\mathrm{shift}}},r_{{\mathrm{down}}} \pm r_{{\mathrm{shift}}}} \right] \times n}$ and the vector corresponding to the sub-image as ${\mathbf{t}}_{0,j}^{\left[ {r_{{\mathrm{up}}},r_{{\mathrm{down}}}} \right]} = {\mathbf{t}}_{i,j}^{\left[ {r_{{\mathrm{up}}} \pm r_{{\mathrm{shift}}},r_{{\mathrm{down}}} \pm r_{{\mathrm{shift}}}} \right]}$, where *r*_shift_ is the number of rows shifted (assuming integer row-shifting for convenience). Thus, the forward imaging model of Eq. (2) can be transformed into a convolution version to be efficiently solved (see Eq. (S1)).

### System calibration

According to the model and algorithm described above, only the encoding kernel corresponding to the on-axis point source needs to be calibrated. Thus, capturing one pattern generated by the on-axis point source is enough for determining the light-field transmission property of the diffuser, without the requirement of any optical parameters of the system. On the other hand, the pattern is combined by the sub-images corresponding to approximately non-overlapped sub-beams emitted angularly by the point source. Non-overlapped light-beam dividing is an ideal situation, and adjacent sub-images in practice partially overlap. If the support of the sub-image is not too small compared to the whole support of the pattern, the sub-image can be considered to have a relatively large centre region independent of adjacent sub-images, so that the corresponding sub-beam can be reasonably approximated to not overlap with others. Consequently, the captured pattern can be evenly segmented into a series of non-overlapping sub-images with an appropriate sampling rate (see Fig. S2). Finally, the objective light field can be computationally reconstructed from a captured image by incorporating the calibrated encoding kernel into the optimization procedure of Eq. (3), achieving single-exposure lensless light-field imaging through diffuser encoding.

### System setup

The lensless imaging system was constructed using a commercially available holographic diffuser (Edmund, Polycarbonate, 0.5°) and an sCMOS sensor (PCO.edge 4.2, resolution: 2048 × 2048 pixels, pixel size: 6.5 × 6.5 µm^2^). The diffuser was placed at a distance of 10 mm in front of the sensor to generate high-contrast pseudorandom patterns. A 6 × 6 mm^2^ square aperture was located close to the diffuser to limit the support of the pattern. In addition, a halogen lamp together with a 15 µm pinhole was used to produce a point source illumination. This point source was placed 20–50 mm away from the diffuser and adjusted to generate a pattern located at the centre of the sensor. In this case, the point source was approximated as being on the axis. A computer (CPU: i7-7700K, RAM: 64 GB) and MATLAB programs^33^ without parallel computing were used to carry out the decoupling reconstruction.

## Supplementary information

Supplementary Infomation
