# Supplementary material for: Lensless light-field imaging through diffuser encoding
Source: Light Sci Appl. 2020 Aug 19;9:143. doi: 10.1038/s41377-020-00380-x (PMC7438505; doi:10.1038/s41377-020-00380-x)
Supplement: Supplementary file 1 — Supplementary Infomation [file 41377_2020_380_MOESM1_ESM.pdf]

Supplementary Information for

Lensless light-field imaging through diffuser encoding

Zewei Cai[1,2], Jiawei Chen[2], Giancarlo Pedrini[1], Wolfgang Osten[1], Xiaoli Liu[2,*], Xiang Peng[2,*]

1 Institut für Technische Optik, Universität Stuttgart, Pfaffenwaldring 9, 70569 Stuttgart, Germany

2 College of Physics and Optoelectronic Engineering, Shenzhen University, Shenzhen Guangdong 518060, China

Correspondence: E-mail: lxl@szu.edu.cn (Xiaoli Liu); xpeng@szu.edu.cn (Xiang Peng)

System modeling

Fig. S1 shows the structure of the system model determined by Eq. (1) and Eq. (2) for the case n = 16. The matrices $\mathbf{H}_i = \left(\mathbf{t}_{i,1}, \mathbf{t}_{i,2}, \cdots, \mathbf{t}_{i,n}\right) \in \Re^{m \times n}$ and $\mathbf{T} = \left(\mathbf{H}_1, \mathbf{H}_2, \cdots, \mathbf{H}_k\right) \in \Re^{m \times nk}$ describe the properties of the single-point and ensemble light-field transmission, respectively. The number of rows of **T** is equal to the number of pixels used, m. The number of columns (number of light rays) is given by the numbers of angular and spatial samplings, n and k. The angular and spatial samplings are equivalent to the angular and spatial resolutions of the light field, i.e., $n = N_u \times N_v$ and $k = N_s \times N_t$. The vector $\mathbf{t}_{i,j}$ is related to a specific segment of the pseudorandom pattern, namely a sub-image corresponding to the j-th light ray of the i-th point source, as illustrated by the cyan connecting lines and corresponding yellow boxes in Fig. S1. According to the random roughness distribution and the lateral shift invariance of the diffuser, the sub-images are different from each other, making the vector $\mathbf{t}_{i,j}$ linearly independent.

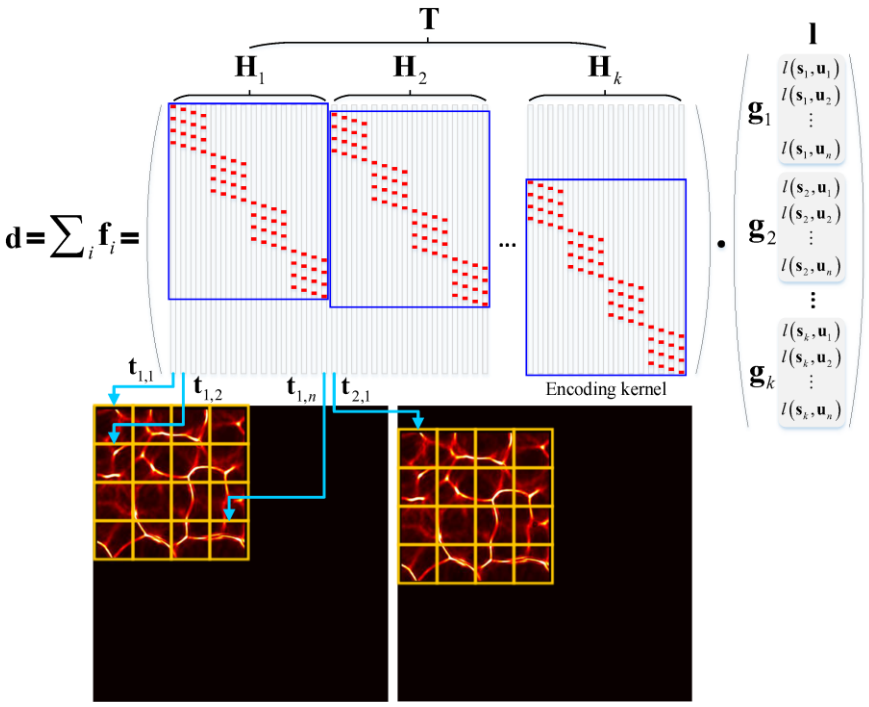

Fig. S1. Schematic diagrams of the system modeling of lensless light-field imaging through diffuser encoding.

System calibration

Fig. S2 shows the calibration procedure by using an on-axial point source p_0 to generate a pseudorandom pattern. The pattern is evenly segmented to a series of non-overlapped sub-images, which exactly correspond to the column vectors of the encoding kernel, i.e., $t\left(\mathbf{x}; \mathbf{0}, \mathbf{u}\right)$.

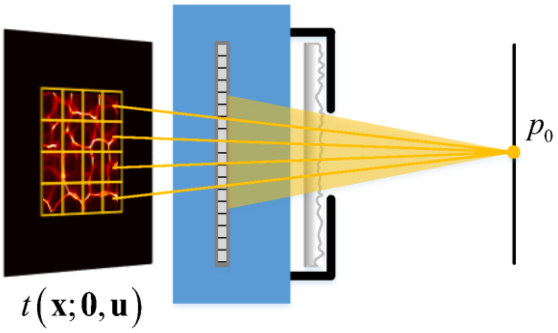

Fig. S2. System calibration.

Setup

The aperture close to the diffuser limits the imaging region on the sensor. The intensity in such a region generates a support of the detected pattern, as shown by the orange box in Fig. S3a. When the point source is shifted laterally, the pattern correspondingly moves on the sensor. The region outside the support determines the maximum lateral spacing of point sources, as shown in Fig. S3b. Furthermore, the size of the support depends on the distance from the point source to the diffuser, i.e., the depth. As shown in Fig. S3c, when the point source is closer to the system, the support becomes larger, while the lateral spacing reduces. This means that the field of view (FoV) becomes smaller.

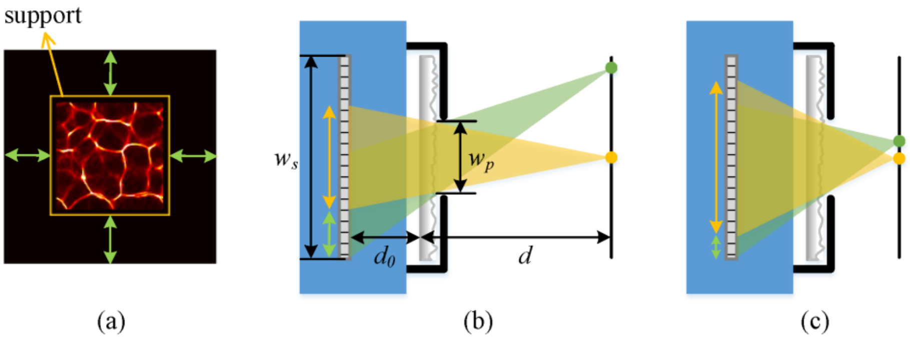

Fig. S3. System details: (a) support of the pattern; (b) relation between FoV and aperture; (c) relation between FoV and depth.

According to Fig. S3b, the FoV is

$$\mathrm{FoV} = \frac{1}{d_0}\left[w_s d - w_p\left(d + d_0\right)\right], \tag{S1}$$

where w_s is and w_p are the sizes of the sensor and the aperture, respectively, d_0 is the distance from the diffuser to the sensor, and d is the depth. If the system is fixed, w_s and d_0 are constants. For a specific w_p, the FoV is proportional to d, which is consistent with the above discussion. For a specific d, the FoV can be increased by decreasing w_p. However, a small aperture will reduce the amount of

light and thus may require longer exposure time. Besides, a small support may also limit the angular sampling on the pattern.

The size of the aperture can be selected according to the desired FoV and the depth range. In the experiments, we used an aperture with a size of 6 × 6 mm^2. Then 12 patterns were captured by axially moving the point source far away from the system with steps of 2 mm. Fig. S4a shows three patterns (Pattern 1, 6, and 12), captured at the distances: 25 mm, 35 mm, and 47 mm, respectively. It can be seen that the size of the patterns decreases with increasing distance, while the shape of the patterns remains unchanged. According to Eq. (S1), the FoVs related to Pattern 1 and Pattern 12 are 12.28 mm and 28.36 mm, respectively. The requirement of the experiments are thus satisfied.

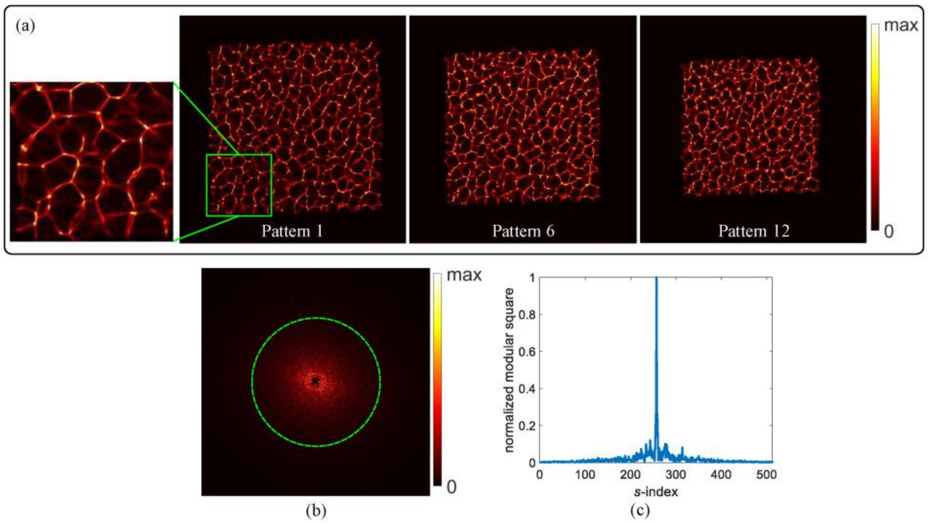

Fig. S4. (a) Pseudorandom patterns obtained by axially moving the point source; (b) spectrum of Pattern 1; (c) central cross-section of the spectrum.

The enlarged view (see left side of Fig. S4a) shows the high-contrast structure of Pattern 1. The structure remains in a large depth range. Fig. S4b and S4c show the spectrum and its central cross-section to Pattern 1, respectively. In Fig. S4b, the central region is suppressed and the green dashed circle indicates the valid spatial frequencies. The system can record high-frequency information, therefore, high-resolution light fields can be reconstructed, avoiding the resolution limitation of the sensor.

Spatio-angular sampling

Once a pattern is chosen as a base, it can be segmented for the decoupling reconstruction according to the desired spatio-angular resolutions. First, a spatial resolution can be selected. Since the resolution of the camera used is 2048 × 2048 pixels, the spatial resolution can be conveniently adjusted by a

multiple of 2 × 2. Thus, spatial samplings like 1024 × 1024, 512 × 512, and 256 × 256 are used. Similarly, other spatial samplings, such as 1280 × 1280, 640 × 640, and 320 × 320, can also be used. Subsequently, the captured pattern can be scaled with the selected spatial resolution. Fig. S5a shows the scaled Pattern 6 with a spatial sampling of 512 × 512. Then, the support of the scaled pattern can be identified. This can be easily processed by horizontally and vertically projecting the pattern, as shown by the curves in Figs. S5b and S5c, respectively. Finally, the sub-images can be obtained by evenly segmenting the support. The size of each sub-image can be roughly determined based on the full size of the support and the desired angular resolution. For example, by using a size of 60 × 60 pixels, 6 × 6 sub-images are obtained, as shown in Fig. S5d. Therefore, the spatio-angular resolution used for light-field reconstruction is 512 × 512 × 6 × 6. Note that there is no special requirement for the selection of the spatio-angular sampling.

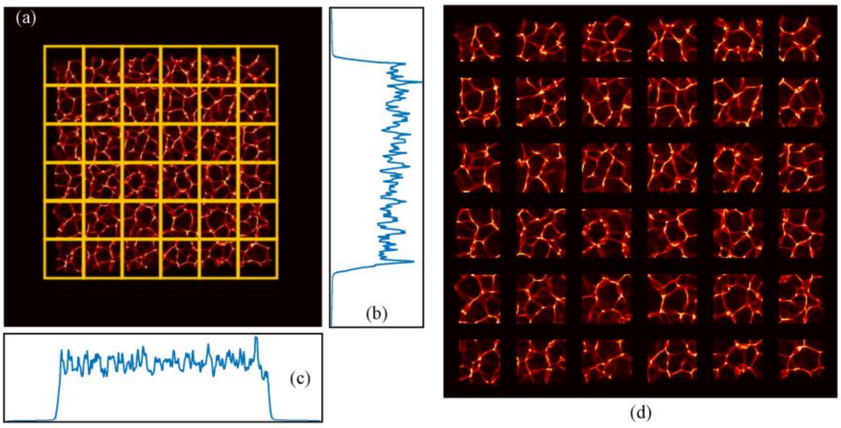

Fig. S5. Spatio-angular sampling: (a) scaled Pattern 6 with the spatial sampling of 512 × 512; (b) – (c) horizontal and vertical projections of (a), respectively; (d) 6 × 6 sub-images.

Simulation scene of multiple sparse object points

Fig. S6 shows an example of lateral shifting. The support of each captured pattern, except for Pattern 6 that was used as a base for calibration, was randomly shifted (integer pixels shift). According to the shift invariance property of the diffuser, the shifting of the pattern is equivalent to the lateral translation of the corresponding point source. The point sources used to generate these patterns were locate at different distances from the diffuser. Therefore, the combination of these shifted patterns can be regarded as the intensity detection of a simulated scene consisting of multiple object points located at different positions in the measured volume.

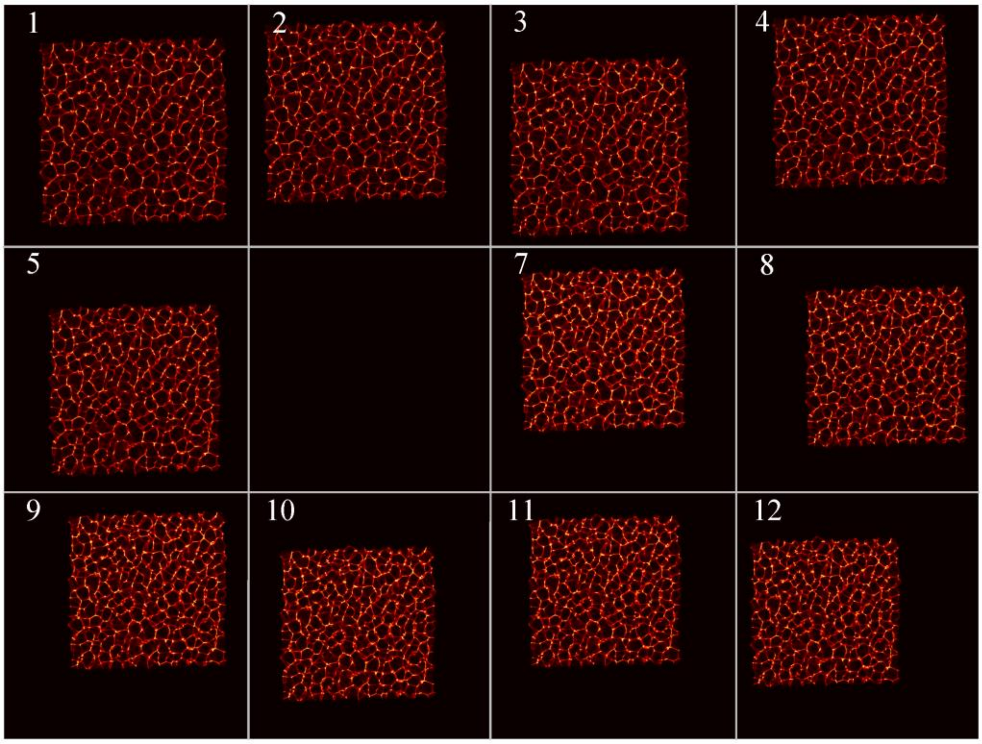

Fig. S6. Lateral shifting of captured patterns.

Light-field reconstruction using a scaled pattern

Light fields related to area objects can be reconstructed correctly as long as a pattern generated by a point source located at the depth near the objects is used. When we used Pattern 1 as a base to reconstruct the light field in the spatio-angular sampling of $1024 \times 1024 \times 6 \times 6$ from the raw image shown in Fig. 5a, an acceptable result could be obtained. This is illustrated by the in-focus slices of the reconstructed light field (see Fig. S7a). Alternatively, we scaled Pattern 6, as shown in Fig. S7b. The enlarged views of Pattern 1 and the scaled pattern show that their structures are basically the same. Interestingly, the light field could also be correctly reconstructed by using the scaled pattern, as illustrated by corresponding in-focus slices in Fig. S7b.

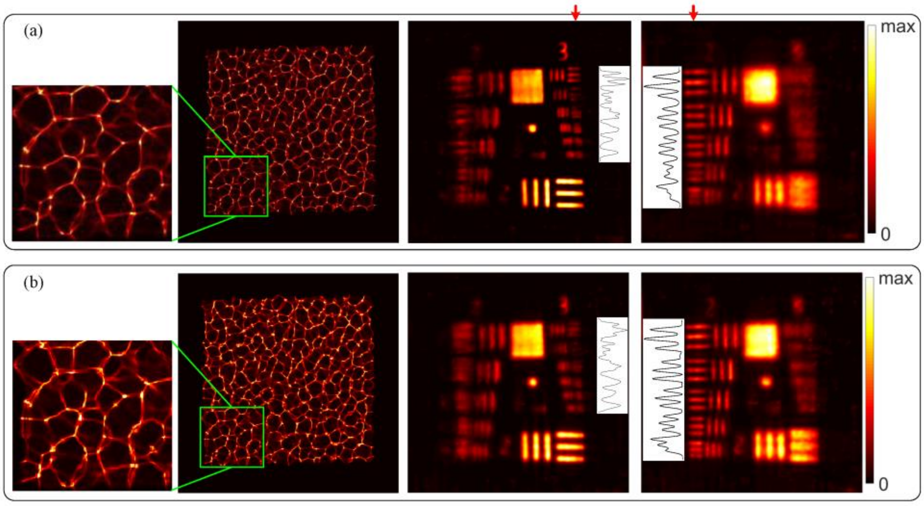

Fig. S7. Light-field imaging of the area object of Fig. 5: (a) Pattern 1 and in-focus slices of the reconstructed light field; (b) scaled version of Pattern 6 and in-focus slices of the reconstructed light field.

Experiment comparison with different iterations

Fig. S8 shows a comparison of reconstructions obtained with different number of iterations. More iterations can suppress artifacts but the reconstructions are blurred.

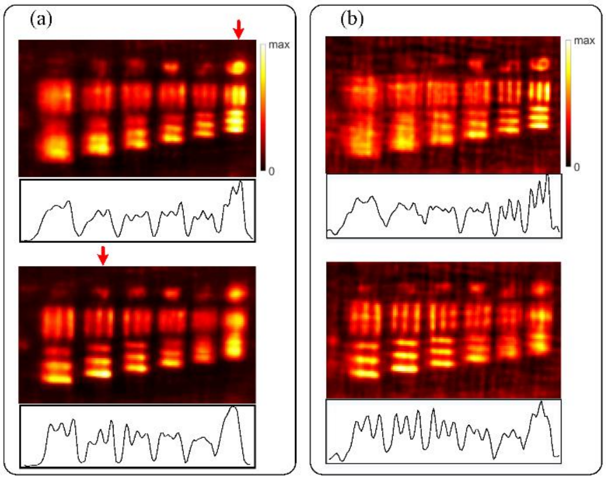

Fig. S8. Comparison results of the in-focus slices concerning the experiment in Fig. 4 with (a) 500 iterations and (b) 200 iterations, both in the spatio-angular sampling of $512 \times 512 \times 6 \times 6$.

Optimization algorithm

Based on the shift invariance of the encoding kernel, the forward imaging model described by Eq. (2) can be replaced with a convolution, similar to that used by Antipa et al.,[1] such that

$$\begin{aligned} d(\mathbf{x}) &= \sum_{\mathbf{u}}\sum_{\mathbf{s}} l(\mathbf{s},\mathbf{u})\, t(\mathbf{x};\mathbf{s},\mathbf{u}) \\ &= \sum_{\mathbf{u}}\sum_{\mathbf{s}} l(\mathbf{s},\mathbf{u})\, t(\mathbf{x}+a\mathbf{s};\mathbf{0},\mathbf{u}), \\ &= \mathbf{C}\sum_{\mathbf{u}} l\left(-\frac{\mathbf{x}}{a},\mathbf{u}\right) * t(\mathbf{x};\mathbf{0},\mathbf{u}) \end{aligned} \qquad \text{(S2)}$$

where t is the sub-image intensity at pixel $\mathbf{x} = (x, y)^{\mathrm{T}}$, corresponding to a unit-radiant light ray with spatio-angular coordinates (**s**, **u**), and d is the detected intensity by summing over the contribution components of all light rays in the detectable field-of-view. A lateral shift of the on-axis point source by **s** results in a translation of the corresponding pattern by a**s**, where a is the magnification. Thus, the sub-image intensity corresponding to the point source located at **s** is given by $t(\mathbf{x};\mathbf{s},\mathbf{u}) = t(\mathbf{x}+a\mathbf{s};\mathbf{0},\mathbf{u})$, and the detected intensity is represented by a two-dimensional discrete convolution (denoted as * in Eq. (S2)) over **x** with a cropping operation **C**. The procedure described by Eq. (3) can be efficiently performed by using the convolution of Eq. (S2) along with appropriate optimization algorithms, such as the alternating direction method of multipliers, which was argued to be suited for distributed convex optimization[2].

1 Antipa, N. *et al.* DiffuserCam: lensless single-exposure 3D imaging. *Optica* **5**, 1-9 (2018).

2 Boyd, S. *et al*. Distributed optimization and statistical learning via the alternating direction method of multipliers. *Foundations and Trends in Machine Learning* **3**, 1–122 (2010).
